# Early-onset renal cell carcinoma in *PTEN* harmatoma tumour syndrome

**DOI:** 10.1038/s41525-020-00148-7

**Published:** 2020-09-29

**Authors:** Raymond H. Kim, Xiangling Wang, Andrew J. Evans, Steven C. Campbell, Jane K. Nguyen, Kirsten M. Farncombe, Charis Eng

**Affiliations:** 1 Fred A. Litwin Family Centre in Genetic Medicine, Familial Cancer Clinic, Princess Margaret Cancer Centre, University Health Network, Department of Medicine, University of Toronto, Toronto, ON Canada; 2 Center for Personalized Genetic Healthcare, Cleveland Clinic Community Care and Population Health, Cleveland, OH USA; 3 Genomic Medicine Institute, Cleveland Clinic Lerner Research Institute, Cleveland, OH USA; 4 Department of Nephrology, Glickman Urological and Kidney Institute, Cleveland Clinic, Cleveland, OH USA; 5 Department of Molecular Medicine, Cleveland Clinic Lerner College of Medicine, Cleveland, OH USA; 6 Laboratory Medicine Program, Department of Pathology, University Health Network, Toronto, ON Canada; 7 Department of Urology, Glickman Urological and Kidney Institute, Cleveland Clinic, Cleveland, USA; 8 Department of Surgery, Cleveland Clinic Lerner College of Medicine, Cleveland, OH USA; 9 Department of Anatomic Pathology, Robert J. Tomsich Pathology and Laboratory Medicine Institute, Cleveland Clinic, Cleveland, OH USA; 10 Toronto General Hospital/Research Institute, University Health Network, Toronto, ON Canada; 11 Department of Genetics and Genome Sciences, and Germline High Risk Cancer Focus Group, Case Comprehensive Cancer Center, Case Western Reserve University School of Medicine, Cleveland, OH USA

**Keywords:** Cancer genetics, Renal cell carcinoma, Cancer screening

## Abstract

Individuals with PTEN hamartoma tumour syndrome (PHTS), including Cowden syndrome (CS), are susceptible to multiple benign hamartomas and an increased risk of cancer, particularly breast, endometrial, and thyroid. As a result, individuals undergo enhanced surveillance for early detection of these cancers. However, less commonly occurring cancers, such as colorectal and kidney, have insufficient guidelines for early detection. Currently, screening for kidney cancer via renal ultrasound begins at 40 years of age, because there were only rare cases of elevated risk in prospective series under 40. There have, however, been accumulating reports of kidney cancer in individuals with CS in their 30s, illustrating a need to lower the age of surveillance. We present additional evidence of renal cell carcinoma in two individuals with CS in their early twenties, and propose a reassessment of the abdominal surveillance in patients with PHTS. We propose biannual screening for kidney cancer beginning at 20 years of age.

## Introduction

PTEN hamartoma tumour syndrome (PHTS) is an umbrella molecular diagnostic term for a subset of disorders where individuals harbour germline *PTEN* (MIM 601728) pathogenic/likely pathogenic variants (collectively referred as pathogenic variants herein) and diverse clinical diagnoses including Cowden syndrome (CS), Bannayan–Riley–Ruvalcaba syndrome, Proteus or Proteus-like syndrome, adult Lhermitte–Duclos disease, and autism spectrum disorders with macrocephaly^1–4^. *PTEN* regulates broad cell processes including cell growth and apoptosis, with inactivation or deletion of this gene leading to development and progression of tumours^5,6^. CS (MIM 158350), a major clinical syndrome umbrellaed as PHTS, is an inherited, autosomal-dominant condition that predisposes individuals to multiple benign hamartomas^7^. There are increased risks of breast (up to 85% lifetime risk in women), thyroid (38%) endometrium (28%), kidney (34%), and colorectal (9–17%) cancers, as well as melanoma (6%) in individuals with PHTS^8–10^. Due to the increased risks of developing specific cancers, patients with PHTS undergo regular enhanced surveillance. This includes thyroid ultrasound, colonoscopy, abdominal ultrasound, and dermatological examination in all adults, with the addition of breast and endometrial surveillance in women^1^.

The original diagnostic criteria for CS were developed by the International Cowden Consortium, whereby individuals must meet a certain number of pathognomonic, major and minor criteria^2^. Based on subsequent accumulating evidence, the operational criteria were reviewed and modified to include the addition of endometrial cancer as a major criterion and renal cell carcinoma (RCC) as a minor criterion in 2000^11^. Surveillance is recommended for all individuals with known germline *PTEN* pathogenic variants, and those with a clinical diagnosis of CS^11^. This was updated by the National Comprehensive Cancer Network (NCCN), where updated guidelines and are published based on continuously modified criteria^12^. Kidney cancer is not as prominent a PHTS-associated malignancy, but three cohort studies identify a 34% lifetime risk with rising risk beginning at 40^13–15^. Patients most frequently present with unilateral tumours between 40 and 50 years of age with papillary (types I and II) and chromophobe types of RCC^13^. Present guidelines by NCCN recommend surveillance via renal ultrasound every 1–2 years starting at 40 years of age for detection of PHTS kidney cancer^12^.

The most well-characterized prospective cohort study from the Cleveland Clinic identified 9/219 patients with a germline *PTEN* pathogenic variant and a history of RCC^16^. Average age at diagnosis was 45.4 years of age, with a median age of 49 years and a 2:1 female-to-male ratio, demographics differing from the reported SEER data for kidney, and renal pelvis cancer (2004–2008) where mean age is 64 and an inverted 2:1 male-to-female ratio^16^. Lifetime risk for developing RCC was estimated to be >30-fold higher^16^. Compared to many other hereditary kidney cancer syndromes, there are many different histologic subtypes present in CS–RCC, whereas the most common type of RCC in the general population (clear cell) is the least common in CS^17^.

Here, we report two cases of early-onset CS–RCC, adding to the mounting evidence of early-onset RCC to consider improving guidelines for clinical diagnosis and time at initiation of renal surveillance.

## Results

### Case report patient 1

#### Patient 1

**Fig. 1:**
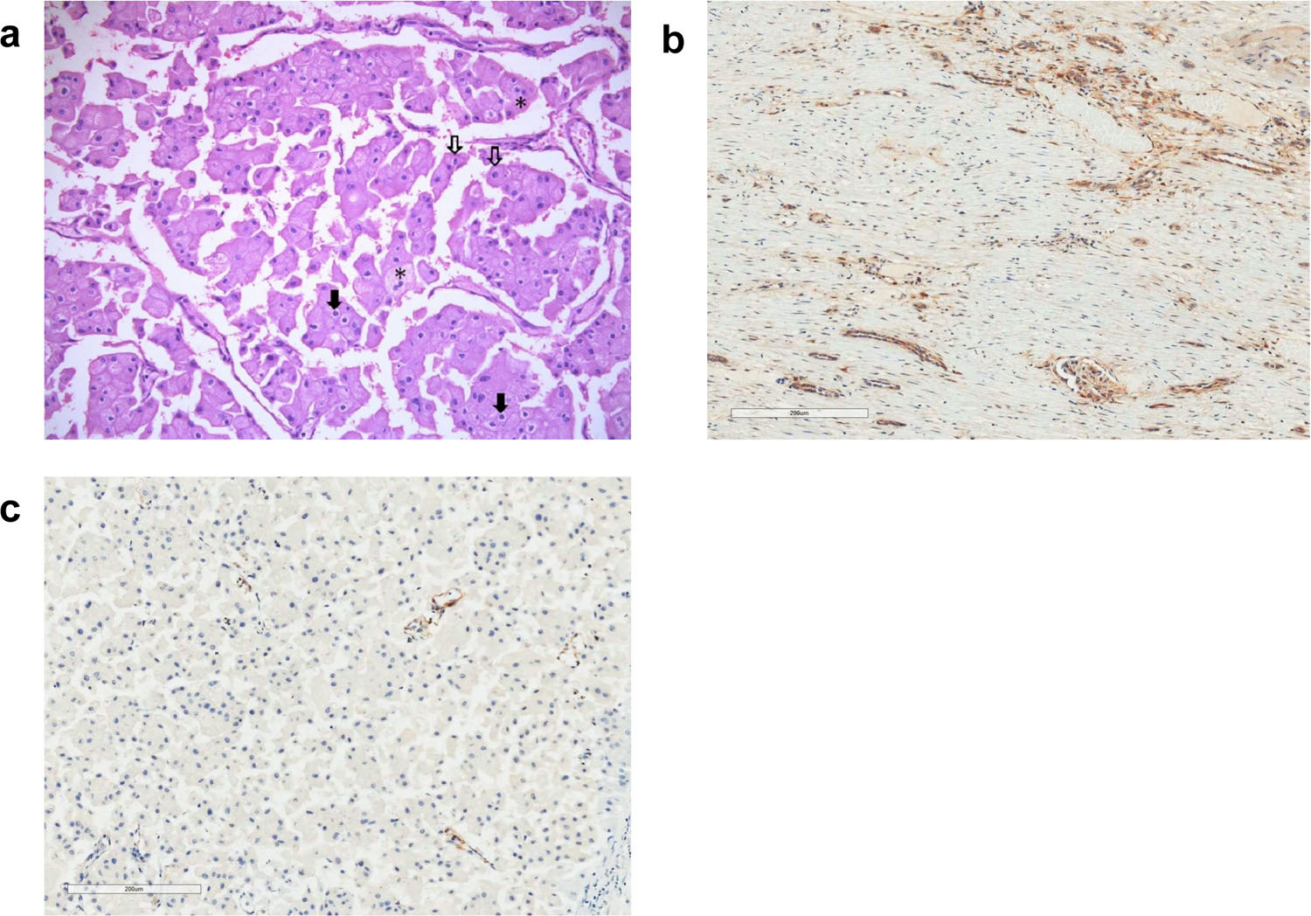
Patient 1 PTEN immunohistochemistry. **a** H&E section of the 11.7 cm right renal mass showing typical features of chromophobe renal cell carcinoma, including cells with prominent cell membranes (open arrows), abundant pale eosinophilic cytoplasm, and perinuclear haloes (solid arrows). Scattered binucleated cells are also present (asterisks) (×200 magnification). **b** PTEN immunohistochemical staining showing positive immunoreactivity in glomeruli, tubules, and endothelial cells of small blood vessels in atrophic renal cortex adjacent to the tumour (×200 magnification). **c** PTEN immunohistochemical staining showing negative immunoreactivity in the tumour cells in contrast to the expected positive staining shown by blood vessels within the tumour (×200 magnification).

A Chinese male presented at the age of 22 with flank pain and was found to have a large right upper pole renal mass on abdominal CT. A right radical nephrectomy was performed following preoperative embolization. The pathology revealed a 11.7 cm chromophobe RCC (see Fig. 1a). The tumour was limited to the kidney (pT2b), and regional lymph nodes were not accessed (pNX). No sarcomatoid features, rhabdoid features, or lymphovascular invasion were identified. The surgical margins were negative for malignancy. Embolization material was found in the renal artery and its branches, along with focal tumour necrosis.

Due to his young age, a hereditary kidney cancer gene panel was conducted (see “Methods”), which revealed a pathogenic variant in the *PTEN* gene (c.388C>T p.Arg130*). He was then referred to the cancer genetics clinic at the University Health Network (UHN) (Toronto, ON) for further assessment. His history revealed that at 12 years of age, he had a follicular adenoma with papillary hyperplasia on thyroid ultrasound. A right hemithyroidectomy was conducted, and although there were features of a papillary tumour, a lack of characteristic nuclear features prevented a diagnosis of papillary carcinoma. Thyroid stimulating hormone was inhibited, and the patient was given a diagnosis of subclinical hyperthyroidism. Physical examination revealed a head circumference of 62 cm, height of 5′8, weight of 261 lbs, no papules in the mouth, and no palmoplantar keratosis. Despite not meeting diagnostic criteria for PHTS based on his history of one major criterion (macrocephaly), and two minor criteria (thyroid lesions and RCC), a diagnosis of CS was made based on the *PTEN* pathogenic variant. He did not have a family history of PHTS. His maternal grandmother was affected with kidney and thyroid disease and his mother has a hyperactive thyroid. All family members declined genetic testing for the *PTEN* variant.

Following his CS diagnosis, PTEN immunohistochemistry was performed on the renal tumour. As shown in Fig. 1b, PTEN staining was negative in the tumour cells, as compared to positive staining in the blood vessels within the tumour (which served as a positive control). He is undergoing yearly surveillance, which revealed a left thyroid nodule (2.2 cm) found to be benign on fine needle aspiration (FNA). He will begin surveillance for other organs at the appropriate age.

### Case report patient 2

#### Patient 2

A 21-year-old white female presented to the PTEN Multidisciplinary Clinic at the Cleveland Clinic for a diagnosis of PHTS. At the age of 19 years, she underwent *PTEN* genetic testing due to a history of hemangiomas, hemihyperplasia, and developmental delay. She was found to have a pathogenic *PTEN* missense variant c.464A>G (p.Tyr155Cys). She did not have a family history of PHTS. Her mother underwent thyroidectomy due to goitre at the age of 17 years and her sister has Hashimoto’s thyroiditis. Her maternal grandfather had colectomy due to more than 100 polyps and his brother had stomach cancer. All family members have not pursued genetic testing for the *PTEN* variant. During her visit, she reported chronic fatigue but denied gross haematuria or flank pain. Physical exam disclosed left hemihyperplasia, macrocephaly with a head circumference of 61 cm, papillomatous papules over her facies, and large vascular malformations over her torso and the left lower extremity. There were no clinical manifestations of palmar pits, scrotal tongue, or gingival papillosis. A thyroid ultrasound showed enlarged thyroid with several nodules. FNA was performed and it revealed benign follicular nodules. She was also found to be anaemic with a Hgb of 8.5 g/dL. Iron studies revealed iron deficiency with ferritin 33.2 ng/mL, iron 16 μg/dL, and transferrin saturation 5%. Colonoscopy revealed multiple hamartomatous (ganglioneuroma) polyps. Iron supplementation was initiated and the patient responded well with an increase of Hgb to 10.3 g/dL within 10 months.

**Fig. 2:**
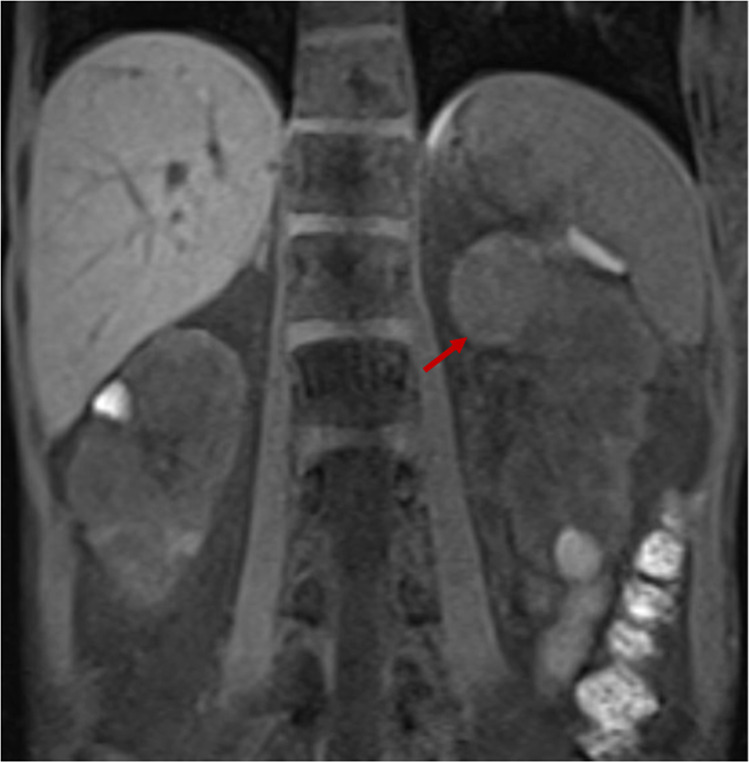
Patient 2 imaging. MRI of kidneys showing bilateral neoplasms with the largest (arrow) in the medial aspect of the upper pole of the left kidney.

**Fig. 3:**
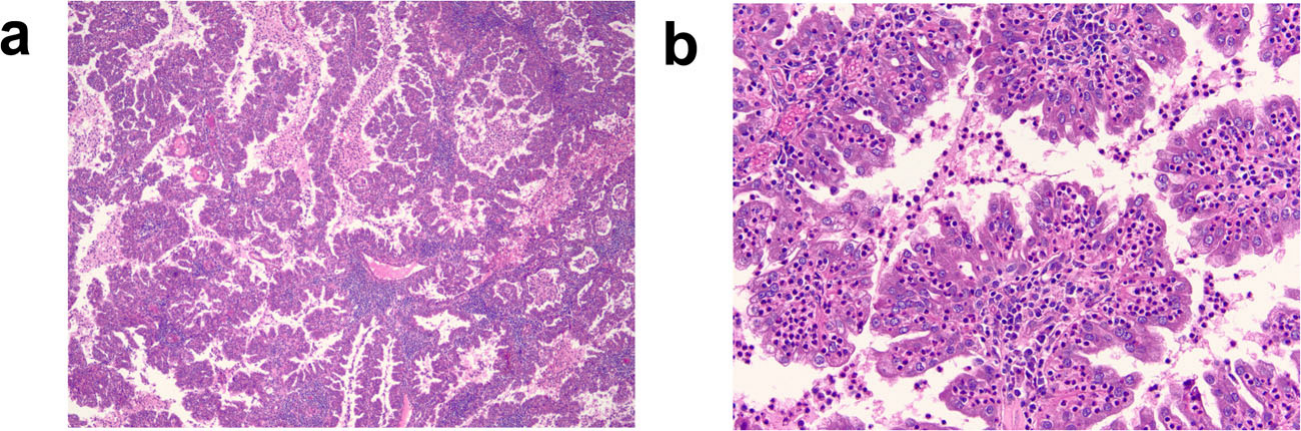
Patient 2 pathology. **a** H&E section of the 4.5 cm left renal mass showing a papillary renal cell carcinoma with fibrovascular cores filling a cystic space and composed of eosinophilic cytoplasm (×4 magnification). **b** Higher magnification highlights readily identifiable prominent nucleoli, ISUP grade 3 (H&E, ×20 magnification).

Prior to her visit, the patient had a computed tomography angiography (CTA) of the abdomen at her local hospital due to known vascular malformations and revealed multiple simple cysts within both kidneys but no other pathology. Within 8 months of her initial CTA, the patient experienced painless gross haematuria and a magnetic resonance angiography of the abdomen was performed. This revealed multiple lesions in the kidneys with the largest in the medial aspect of the upper pole of the left kidney measuring 3.2 cm in greatest dimension, suggestive of RCC versus hamartomas. A cystoscopy was performed, and was normal. Within 13 months, a subsequent MRI of the kidneys showed bilateral enhancing renal neoplasms with interval enlargement of the dominant 4.5 cm heterogeneously enhancing lesion in the upper pole of left kidney that demonstrates restricted diffusion, likely papillary subtype RCC (Fig. 2). A left partial nephrectomy was performed and pathology revealed RCC, papillary PHTS-associated type, ISUP grade 3 (see Fig. 3)^16^. The tumour extended into the pelvicalyceal system with negative margins of resection. Post-operatively, the patient has been doing well clinically and continues with close monitoring of the other lesions within both kidneys as well as age-appropriate surveillance for the other PHTS-relevant organs.

## Discussion

Although kidney cancer is a minor criterion for diagnosis of CS, it is not considered a frequently occurring feature. Genotype–phenotype correlations are not consistently reported in CS, with reports identifying specific germline *PTEN* pathogenic variants in PHTS cancers, particularly: frameshift variants in thyroid cancer^18^, nonsense variants in colorectal cancer^9^, promoter variants and breast cancer^9^, as well as missense variants in autism spectrum disorder^19^. However, other studies have found no associations with specific variants/protein regions and cancer types^8,20^, suggesting additional factors may contribute to PHTS phenotypes^1^.

Results from a prospective cohort study on nine patients with RCC and a *PTEN* pathogenic variant at the Cleveland Clinic uncovered six tumours as papillary RCCs, and two tumours as chromophobe RCCs^16^. Another study conducted at the National Cancer Institute’s (NCI) Centre for Clinical Research identified 4/24 patients (two men and two women) meeting clinical CS criteria and confirmed germline *PTEN* pathogenic variants with a history of RCC^17^. Three individuals were diagnosed with kidney cancer in their 50 s, whereas one woman was 32 years of age^17^. There was no family history of RCC, and two patients had had papillary type I RCC, one patient had clear cell RCC, and the other had bilateral chromophobe RCC^17^. Combining the two studies resulted in the following CS–RCC tumour distribution: 54.5% papillary type I, 18.2% papillary type II, 18.2% chromophobe, and 9.1% clear cell^17,21^.

Lowering the age of kidney cancer screening has been suggested by multiple reports. Cumulative cancer risks were reported following a review of 210 patients who met the accepted diagnostic criteria for CS (90% from published medical literature and 10% from Mayo Clinic records)^22^. Based on these results, screening for kidney cancer was recommended to begin at 33 years of age to capture 95% of cases, or at 28 years of age (the youngest reported case) to capture 100% of cases^22^. Lowering the age of surveillance to 30 years of age for CS–RCC has been suggested in more recent reports as well^8,13^. A study at the Institut Bergonié Genetic Laboratory (Bordeaux, France) identified 146 individuals with deleterious germline *PTEN* pathogenic variants and detailed phenotypic information^8^. Two women and one man (2% of participants) developed RCC^8^. From this, the French Cowden Disease Network proposed to begin kidney cancer screening via renal ultrasounds and/or renal MRI at 30 years of age, continued annually if there was a family history of kidney cancer or every two years in the absence of a family history^8^.

Subsequently, an electronic retrospective review of patients seen at Boston Children’s Hospital between 1996 and 2011 identified 34 children under the age of 21 years using the search parameters: *PTEN*, Bannayan–Riley–Ruvalcaba, and CS^23^. All participants had molecular confirmation of PHTS^23^. An 11-year-old male, who had previously developed follicular thyroid carcinoma at 7 years of age, presented with RCC^23^. This is the only known report, to our knowledge, of a confirmed *PTEN* pathogenic variant and RCC in a child^23^. It was hypothesized that rare tumours in these children resulted from combined pathogenic variants in tumour suppressor genes/oncogenes and *PTEN*, but no suggestions for updating surveillance criteria for CS–RCC were given^23^. Finally, an atypical presentation of CS, where the proband did not meet diagnostic or testing criteria, identified four tumours within two years from 31 to 33 years of age^13^. This included acral melanoma, a follicular variant of papillary thyroid carcinoma, and two clear cell RCCs^13^. Exome sequencing on the proband and family identified many variants of interest, including a de novo heterozygous deleterious pathogenic variant in *PTEN* and variants in genes somatically associated with melanoma (*MIB2*) and with clear cell RCC (*CEACAM1*)^13^. Although early diagnosis of clear cell RCC has a good prognosis, these tumours were identified incidentally, as screening for CS would not begin for another decade^13^. Therefore, there was agreement that surveillance for CS–RCC should follow the recommendations of the French Cowden Disease Network and lower screening to 30 years of age in all patients harbouring a *PTEN* deleterious pathogenic variant, regardless of family history^13^.

Surveillance recommendations in other hereditary syndromes at risk of kidney cancer (e.g. von Hippel–Lindau (VHL) disease) are determined by assessing age-specific tumour risk, youngest reported age of onset, expected growth rate, and rate of tumour progressions affecting clinical treatment^24^. Often, there are no international consensus guidelines for the management of hereditary syndromes. For example, in VHL disease, guidelines from various organizations, such as the NCI, VHL Alliance, and Danish VHL recommend screening for RCC with abdominal CT/MRI to begin at 15–18 years of age^25^. This is based on multiple reports of RCC occurring as young as 16 years of age^26,27^, and initiating screening 1 year earlier^25^. Similar recommendations are made for hereditary papillary RCC Birt–Hogg–Dubé syndrome, and hereditary paragangliomas–pheochromocytomas syndromes^25^. Based on these reports in hereditary-RCC syndromes and our current findings, we propose a reassessment of the abdominal surveillance in patients with PHTS.

At present, clinical parameters have not been defined to establish which individuals with CS are at increased risk of developing kidney cancer, and diagnostic criteria pose a problem with identifying atypical cases of PHTS. Current surveillance guidelines outline screening for CS–RCC at 40 years of age via renal ultrasound every 1–2 years^12^, despite reports of earlier onset. In addition, based on an estimated 34% increased lifetime risk of developing CS–RCC, it was suggested biannual renal imaging via ultrasound or MRI should be used^9^. As the hypovascular nature of papillary and solid histology RCC tumours are difficult to detect via renal ultrasound^28^, CT or MRI as an alternative imaging modality may have higher sensitivity for small lesions and variable tumours^16^. In our second case, a series of CT or MRI of abdomen or kidneys showed the RCC developed and grew fairly rapidly into a size requiring surgery within less than 1 year. Although there was one report of RCC in a child of 11 years of age, no other children have been reported, and our series of multiple adults presenting in their twenties provides evidence to lower the current age of renal screening. We suggest the frequency should be at least biannual, beginning at 20 years of age, until additional paediatric cases support further lowering the age of renal screening.

## Methods

### Ethics approval

Written informed consent was obtained for patient 1 and patient 2. Ethical approval was obtained from the UHN Ethics Committee and the Cleveland Clinic Institutional Review Board for Human Subjects Protection (Protocol IRB-8458-PTEN). This report is in accordance with approval from both institutions.

### Molecular genetic analysis

#### Patient 1

Genomic DNA was extracted, and was analysed using next-generation sequencing (NGS) and the UHN’s Hereditary Panel Version 2.0. Genes analyzed include: *FH* (NM_000143.3), *FLCN* (NM_144997.5), *MET* (NM_001127500.1), *MITF* (NM_000248.3), *PTEN* (NM_000314.4), *SDHA* (NM_004168.2), *SDHC* (NM_003001.3), *TP53* (NM_000546.5), *TSC1* (NM_000368.4), *TSC2* (NM_000548.3), and *VHL* (NM_000551.3). Exonic coding regions along with ±10 base pairs of the intronic regions of the genes in the variant panel were included. This process uses SureSelect Target Enrichment hybrid capture followed by paired-end sequencing using an Illumina sequencing platform. Variant calls were determined using UHN’s clinical laboratory genetics custom bioinformatics pipeline with alignment to genome build GRCh37/hg19. Cartagenia Bench Lab NGS version 5 was used to evaluate variants, with a minimal acceptable coverage of greater than 25× for all reported genomic regions. Variant pathogenicity was determined using the ACMG guidelines, and identified as pathogenic, likely pathogenic, uncertain, likely benign, or benign^29^.

A heterozygous *PTEN* variant c.388C>T p.Arg130* (NM_000314.4) causing a premature stop codon 130 was identified as pathogenic^29^. This variant has rarely been reported in general population databases; however, it has been reported as pathogenic in ClinVar by multiple laboratories. It is a loss of function variant that was reported in ClinVar to be associated with CS (RCV000008263.6) and PTEN hamartoma syndrome (RCV000199099.8).

#### Patient 2

PTEN genetic testing was performed in a College of American Pathologists (CAP)-accredited and Clinical Laboratory Improvement Amendments (CLIA)-certified clinical diagnostic laboratory (Invitae Corp., San Francisco, CA). Genomic DNA obtained from this patient was enriched for targeted regions using a hybridization-based protocol, and sequenced using Illumina technology. All targeted regions were sequenced with >50× depth. Reads were aligned to a reference sequence (GRCh37), and sequence changes were identified and interpreted in the context of a single clinically relevant transcript (NM_000314.4). Variants were interpreted and reported according to ACMG guidelines^29^.

A heterozygous *PTEN* variant c.464A>G p.Tyr155Cys was identified as pathogenic. This variant is not present in population databases. It has been reported in individuals affected with PHTS^8,30–32^. Experimental studies have shown that this missense change abolishes the phosphatase activity of the PTEN protein in vitro, which is critical for its tumour suppressor function^32–34^.

### *PTEN* immunohistochemistry

#### Patient 1

Based on the patient’s germline *PTEN* pathogenic variant, *PTEN* immunohistochemical staining was performed at UHN on paraffin sections using a rabbit monoclonal anti-PTEN primary antibody (Lot 138G6, Cell Signaling Technology) at a dilution of 1:50. Renal tumour cells showed negative immunoreactivity for *PTEN* when compared to inflammatory cells, stroma, and small blood vessels, which were positive and acting as internal controls. External controls were appropriate.

### Reporting summary

Further information on research design is available in the Nature Research Reporting Summary linked to this article.

## Supplementary information

Reporting Summary

## Data Availability

The data that support the findings in this study are available upon reasonable request from the corresponding author (R.H.K). The sequencing data that support the findings of this study will be available in ClinVar shortly after publication under the following accession numbers: SCV001430888 (patient 1) and SCV001430889 (patient 2).
