# Supplementary material for: Early-onset renal cell carcinoma in PTEN harmatoma tumour syndrome
Source: NPJ Genom Med. 2020 Sep 29;5:40. doi: 10.1038/s41525-020-00148-7 (PMC7525494; doi:10.1038/s41525-020-00148-7)
Supplement: Supplementary file 1 — Reporting Summary [file 41525_2020_148_MOESM1_ESM.pdf]

nature research

Corresponding author(s): Dr. Raymond Kim; NPJGENMED-00536

Last updated by author(s): Jun 5, 2020

Reporting Summary

Nature Research wishes to improve the reproducibility of the work that we publish. This form provides structure for consistency and transparency in reporting. For further information on Nature Research policies, see our Editorial Policies and the Editorial Policy Checklist.

Statistics

For all statistical analyses, confirm that the following items are present in the figure legend, table legend, main text, or Methods section.

n/a	Confirmed	
☒	☐	The exact sample size (n) for each experimental group/condition, given as a discrete number and unit of measurement
☒	☐	A statement on whether measurements were taken from distinct samples or whether the same sample was measured repeatedly
☒	☐	The statistical test(s) used AND whether they are one- or two-sided<br>*Only common tests should be described solely by name; describe more complex techniques in the Methods section.*
☒	☐	A description of all covariates tested
☒	☐	A description of any assumptions or corrections, such as tests of normality and adjustment for multiple comparisons
☒	☐	A full description of the statistical parameters including central tendency (e.g. means) or other basic estimates (e.g. regression coefficient) AND variation (e.g. standard deviation) or associated estimates of uncertainty (e.g. confidence intervals)
☒	☐	For null hypothesis testing, the test statistic (e.g. F, t, r) with confidence intervals, effect sizes, degrees of freedom and P value noted<br>*Give P values as exact values whenever suitable.*
☒	☐	For Bayesian analysis, information on the choice of priors and Markov chain Monte Carlo settings
☒	☐	For hierarchical and complex designs, identification of the appropriate level for tests and full reporting of outcomes
☒	☐	Estimates of effect sizes (e.g. Cohen's d, Pearson's r), indicating how they were calculated

Our web collection on statistics for biologists contains articles on many of the points above.

Software and code

Policy information about availability of computer code

Data collection	No software was used.
Data analysis	No software was used.

For manuscripts utilizing custom algorithms or software that are central to the research but not yet described in published literature, software must be made available to editors and reviewers. We strongly encourage code deposition in a community repository (e.g. GitHub). See the Nature Research guidelines for submitting code & software for further information.

Data

Policy information about availability of data

All manuscripts must include a data availability statement. This statement should provide the following information, where applicable:

- Accession codes, unique identifiers, or web links for publicly available datasets
- A list of figures that have associated raw data
- A description of any restrictions on data availability

The data that supports the findings in this study are available upon reasonable request from the corresponding author (R.K.). The data are not publicly available as they contain information that could compromise research participant privacy or consent.

Field-specific reporting

Please select the one below that is the best fit for your research. If you are not sure, read the appropriate sections before making your selection.

☒ Life sciences ☐ Behavioural & social sciences ☐ Ecological, evolutionary & environmental sciences

For a reference copy of the document with all sections, see nature.com/documents/nr-reporting-summary-flat.pdf

Life sciences study design

All studies must disclose on these points even when the disclosure is negative.

Sample size	Case report of two individuals. The disease and symptoms we are reporting on are rare, therefore increasing sample size is not feasible.
Data exclusions	No data was excluded.
Replication	Case report of two individuals. Replication of experimental design was not needed.
Randomization	We did not have experimental groups, therefore randomization was not required.
Blinding	Blinding was not relevant to this study. Case report of two individuals.

Reporting for specific materials, systems and methods

We require information from authors about some types of materials, experimental systems and methods used in many studies. Here, indicate whether each material, system or method listed is relevant to your study. If you are not sure if a list item applies to your research, read the appropriate section before selecting a response.

Materials & experimental systems

n/a	Involved in the study	
☐	☒	Antibodies
☒	☐	Eukaryotic cell lines
☒	☐	Palaeontology and archaeology
☒	☐	Animals and other organisms
☐	☒	Human research participants
☒	☐	Clinical data
☒	☐	Dual use research of concern

Methods

n/a	Involved in the study	
☒	☐	ChIP-seq
☒	☐	Flow cytometry
☒	☐	MRI-based neuroimaging

Antibodies

Antibodies used	Rabbit monoclonal anti-PTEN primary antibody (Lot 138G6, Cell Signaling Technology) at a dilution of 1:50.
Validation	Commercially available PTEN antibody was used and the staining was performed in an automated clinical immunohistochemistry lab. For Research Use Only. Not For Use In Diagnostic Procedures. Species reactivity is determined by testing in at least one approved application (e.g., western blot).

Human research participants

Policy information about studies involving human research participants

Population characteristics	Two participants were recruited for this case report. Patient 1 is a Chinese male, 22 years of age, and Patient 2 is a white female, 21 years of age. Patient 1 has a pathogenic variant in the PTEN gene (c.388C>T p.Arg130*). Patient 1 has macrocephaly, thyroid lesions and renal cell carcinoma, leading to a diagnosis of Cowden Syndrome. He is undergoing yearly surveillance and has had a left thyroid nodule. Patient 2 has pathogenic PTEN missense variant c.464A>G (p.Tyr155Cys). Patient 2 has macrocephaly, left hemihyperplasia, papillomatous papules over her facies, and large vascular malformations over her torso and the left lower extremity. She also had thyroid nodules, anemia, and multiple hamartomatous polyps, before diagnosis of renal cell carcinoma. She continues to be monitored with yearly surveillance.
Recruitment	One adult male and one adult female were recruited into this case report based on their diagnosis of PTEN Hamartoma Tumour Syndrome and early-onset renal cell carcinoma (under the age of 40 years of age). There was no recruitment bias, due to low numbers of individuals meeting this criteria.

Ethics oversight	Ethical approval was obtained from the University Health Network (UHN) Ethics Committee and The Cleveland Clinic Institutional Review Board for Human Subjects Protection (Protocol IRB-8458-PTEN)

Note that full information on the approval of the study protocol must also be provided in the manuscript.
